# A hydraulically driven colonoscope

**DOI:** 10.1007/s00464-016-4784-2

**Published:** 2016-07-22

**Authors:** Stuart A. Coleman, Silvia C. Tapia-Siles, Markus Pakleppa, Jan B. Vorstius, Robert P. Keatch, Benjie Tang, Alfred Cuschieri

**Affiliations:** 1 Institute for Medical Science and Technology (IMSaT), University of Dundee, 1 Wurzburg Loan, Dundee, DD2 1FD UK; 2 Centro de Investigaciones en Procesos Industriales, Universidad Privada Boliviana, Cochabamba, Bolivia; 3 Division of Mechanical and Electronic Engineering, University of Dundee, Dundee, UK; 4 Cuschieri Skills Centre, University of Dundee, Dundee, UK

**Keywords:** Colonoscopy, Robotic, Colorectal cancer, Screening, Hydraulic

## Abstract

**Background:**

Conventional colonoscopy requires a high degree of operator skill and is often painful for the patient. We present a preliminary feasibility study of an alternative approach where a self-propelled colonoscope is hydraulically driven through the colon.

**Methods:**

A hydraulic colonoscope which could be controlled manually or automatically was developed and assessed in a test bed modelled on the anatomy of the human colon. A conventional colonoscope was used by an experienced colonoscopist in the same test bed for comparison. Pressures and forces on the colon were measured during the test.

**Results:**

The hydraulic colonoscope was able to successfully advance through the test bed in a comparable time to the conventional colonoscope. The hydraulic colonoscope reduces measured loads on artificial mesenteries, but increases intraluminal pressure compared to the colonoscope. Both manual and automatically controlled modes were able to successfully advance the hydraulic colonoscope through the colon. However, the automatic controller mode required lower pressures than manual control, but took longer to reach the caecum.

**Conclusions:**

The hydraulic colonoscope appears to be a viable device for further development as forces and pressures observed during use are comparable to those used in current clinical practice.

Colonoscopy is the only commonly used method for screening the colon which allows for full endoscopic examination of the colon and for polypectomies and biopsies to be carried out as required. It is recognised as the gold standard for establishing the diagnosis of colorectal cancers, and in excess of ten million colonoscopies are carried out each year [1].

However, the procedure has some limitations. It utilises a conventional colonoscope (CC), which is a flexible endoscope equipped with a steerable tip. Even in expert hands, the passage of the colonoscope up to the caecum can cause pain and discomfort to the patient. For this reason, the procedure is usually carried out under sedation. Concern about pain and discomfort also reduces patient compliance [2]. Advancing the CC through the colon can be technically challenging; proficiency requires long training [3], and errors may lead to adverse events, including iatrogenic colonic perforation. Due in part to the large amount of skilled physician time required to insert the CC, the procedure is relatively expensive and thus it may not be the most cost-effective option for mass screening [4]. Hence, a less painful and semi-automated system may be clinically useful.

Various alternatives to the CC have been trialled with the aim of overcoming its limitations [5]. These range from modifications to the CC [6, 7] to devices which utilise alternative methods of propulsion such as an actively articulated shaft [8], an extending external sleeve [9, 10], inchworm-like motion [11], propulsion by pressurised gas [12] or passive transit through the GI tract [13]. Some of these devices partially automate the procedure and others reduce patient discomfort, but none have successfully replaced the CC [14].

In this paper, we describe the “Hydraulic Colonoscope” (HC), a colonic propulsion system, which aims to reduce patient discomfort and the amount of skill required by the operator to advance the colonoscope to the caecum. The system may reduce discomfort in two ways. Firstly, it is a self-propelled device obviating the need for external pushing; a self-propelled device should reduce forces on the colon and thus patient pain, an outcome which has previously been demonstrated in a clinical study [15]. Secondly, the colon is filled with warm liquid instead of gas; the use of water has been shown to reduce patient discomfort [16], probably because it relaxes the colonic musculature.

The propulsion principle of the HC is for a flexible seal to be formed in the lumen of the colon which is then driven through the colon by pressurised fluid. This principle was used in the Aer-O-Scope [12], which was propelled by pressurised CO_2_; however, the system described in the present report uses water as the pressurised driving fluid. A prototype of the device was constructed and tested in a test bed constructed from porcine colon, reconfigured to simulate human colonic anatomy including flexures and mesenteric attachments. The paper reports on the design of the system and its performance compared to a conventional colonoscope.

## Materials and methods

### System overview

The HC system comprises a colonic vehicle (CV), which is linked to a supporting extra-corporeal system of pumps and valves via a tether. It is controlled and monitored by a control system running on a connected PC.

The body of the CV is surrounded by a balloon, which is able to form a seal within the colonic lumen, blocking fluid flow past the CV. The balloon is flexible and may be inflated or deflated so that it conforms to the varying dimensions of the colonic lumen while maintaining a seal. It is capable of sliding through the lumen because of the low-friction characteristics of the colonic mucosal surface. As the sealing balloon fills the lumen of the colon, the walls of the colon passively guide the CV, and thus the CV will follow the lumen of the colon without an active guidance system. The balloon is linked to an extra-corporeal pressure control system by a 1.8 m long PVC tube, with 6 mm outer diameter. This tube passes through the colon and also acts as a tether, allowing for easy withdrawal of the CV. The tether also passes through a PTFE seal in the anal port, thereby allowing the tether to slide while preventing water leakage from the colon. The mean friction of the tether passing through this seal was measured as 0.35 N (max 0.8 N).

**Fig. 1:**
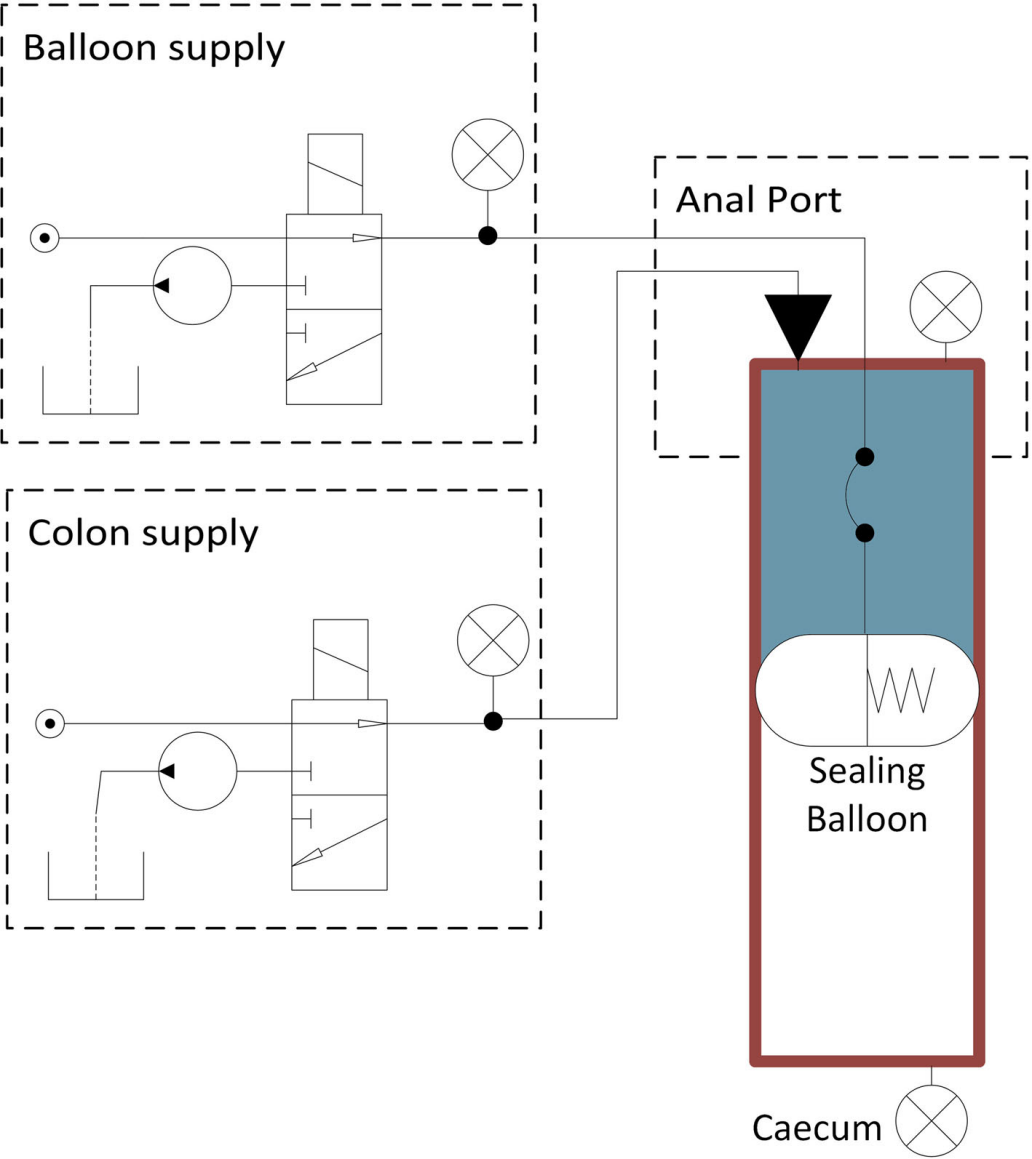
HC system schematic

An extra-corporeal pump system is used to pump water into the colon behind the CV so that a pressure differential is created across the CV. This forces the CV forward through the colon, until it ultimately reaches the caecum. The subsystems for inflating/deflating both the sealing balloon and the colon are similar. Each consists of a pressurised water supply for inflation, a pump for deflation, a solenoid valve for control and a pressure sensor for feedback. A schematic of the elements of the system can be seen in Fig. 1.

Piezoelectric pressure transducers monitor the pressures in the colon in front of and behind the device. Currently, these sensors are attached to the test bed at each end of the colon, but they could be built into the CV and anal port. If excessive water builds up in front of the CV, the balloon is deflated and the entire colon is drained of water.

The CV contains a magnetic tracker (Trakstar Model 180, Ascension Technology Corporation) to allow monitoring of its position and orientation. As the current study is focused on the propulsion system, a camera is not included in the CV; however, a dummy rigid body (Ø 11 mm × 25 mm long) is included at the distal tip to simulate the effects of carrying a camera in the device.

### Control system

The ultimate goal of the control system is to autonomously drive the HC to the caecum, while keeping pressures within the safe physiological range. The control is non-deterministic as the colon’s mechanical properties, constraints and shape vary greatly between individuals and in different sections of a single colon [17, 18].

To achieve this goal, the controller has been designed to integrate two mutually dependent parallel finite state machines (FSMs) in a closed loop. State machines are the oldest known formal model for sequential behaviour [19], where the present state of the system depends on both the previous and current values of the inputs. The transitions between states are made according to rules defined based on experience acquired in previous versions of the HC. For example, if the pressures in the caecum and anus are similar and the CV is not moving, then the controller sends a command to add fluid to the balloon.

**Fig. 2:**
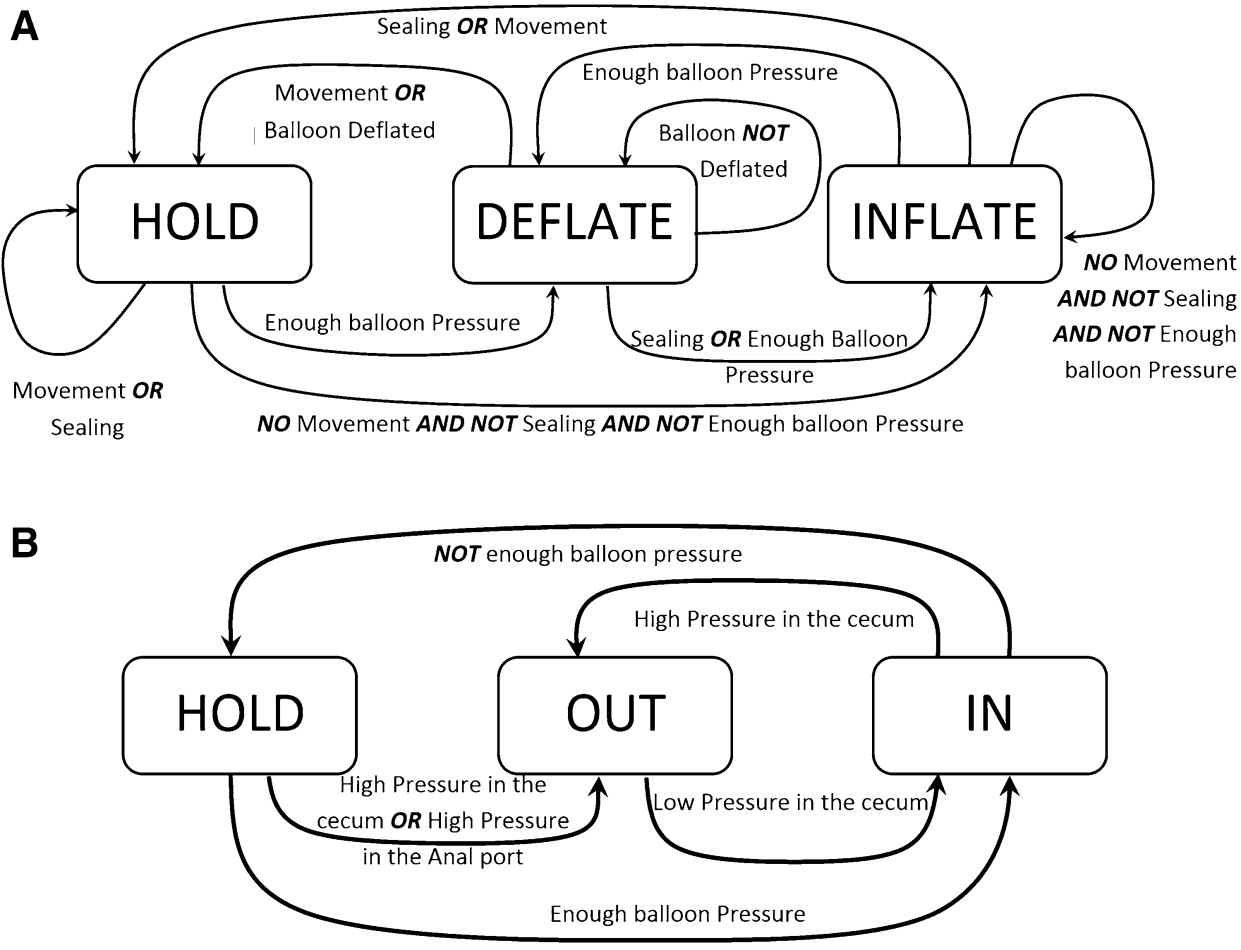
**A** Finite state machine controlling the balloon. **B** Finite state machine controlling water in and out of the colon

The first FSM controls the inflation of the balloons and has three possible states: hold, deflate and inflate (see Fig. 2A). The second state machine controls the insertion or extraction of water from the colon through the anal port (see Fig. 2B). It also has three possible states: hold, pump in and pump out. If the second FSM is set to drain water from the colon, it overrides the first state machine and forces the sealing balloon to deflate in order to drain any water ahead of the CV.

**Fig. 3:**
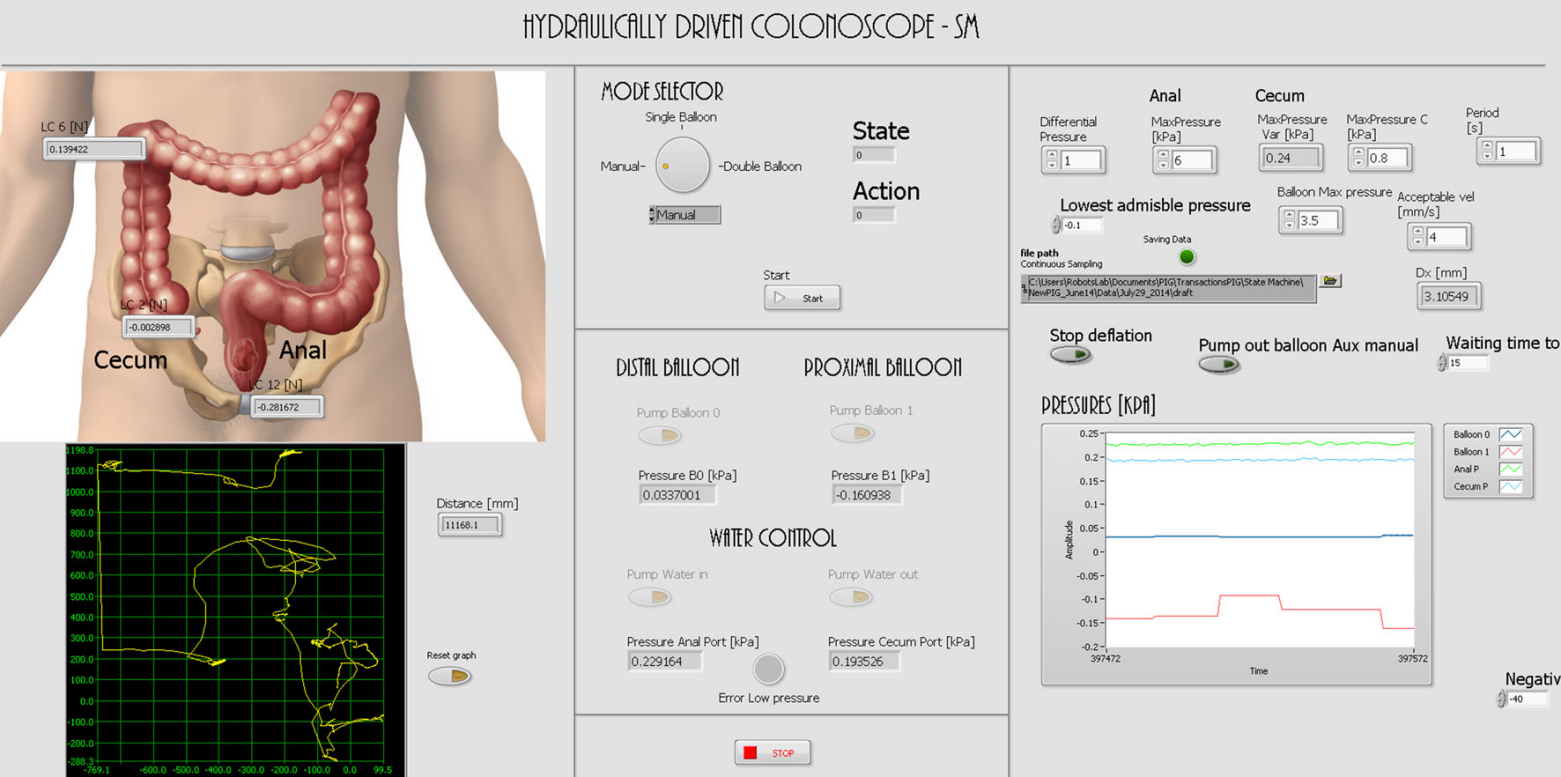
User interface implemented in LabVIEW

The controller allows for either fully automatic or manual control of the HC. During manual control, the operator has access to the information gathered from the sensors via the interface, as well as being able to directly observe the HC and colon. The user interface can be seen in Fig. 3.

The controller has been designed to ensure patient safety by limiting pressures to safe values. These safety measures are included in the system as high-priority interruptions in the different modes of operation. If the pressures being monitored (balloon, caecum and anus) exceed safe values an override mode is entered where an action is taken to reduce pressure. For example, if the maximum admissible pressure in the colon has been exceeded, then the balloon and colon are deflated fully before the FSM system retakes control.

### Test bed

**Fig. 4:**
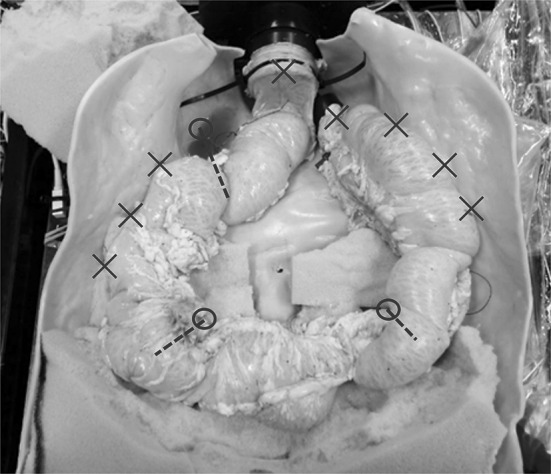
Abdominal cavity cast with porcine colon placed in situ. Fixed attachment points are marked with an “*X*” while load-measuring attachment points are marked with an “*O*”. Note that the test bed also included a cover to further constrain the colon, but this is not shown

The HC system was evaluated by measuring selected parameters to assess the interaction of the device with the colon. Testing was carried out in a porcine colon arranged within a rigid polymer cast of a cadaveric human abdominal cavity and orientated to simulate a patient in the supine position. One end of the colon was attached to a fixed anal port, through which the CV or a CC could be inserted. The other (caecal) end of the colon was attached to a fixed plug. Each end of the colon was attached to a separate pressure sensor. See Fig. 4 for the colon layout.

**Fig. 5:**
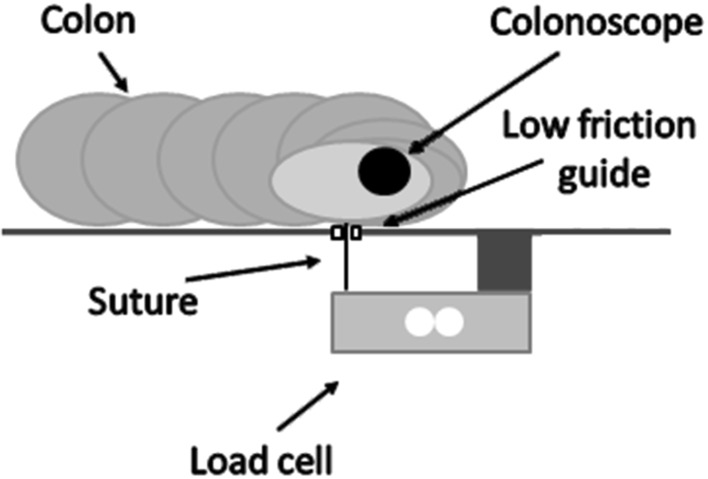
Mesentery attachment point. A suture is attached to the porcine colon to serve as a mesentery. The suture passes through a low-friction guide in the base and is then attached to a load cell which is situated below the base. This constrains the colon and allows forces in the suture to be measured

The colon was held in place by the surrounding abdominal cavity and was attached directly to the walls of the cavity along the ascending and descending colons. It was also constrained by artificial mesenteries placed at the sigmoid, splenic and hepatic flexures. These artificial mesenteries consisted of inelastic cords sutured onto the colon. Each cord passed through a guide and was attached to a load cell fixed on the underside of the test bed (see Fig. 5). This enabled the colon to be constrained in a fairly natural way, while loading on the artificial mesenteries could be measured. The tension in each cord is reduced due to friction where it passes through its guide, causing a small error in measured values. In order to minimise friction, the cord guide is produced from smooth PTFE; errors were measured as <8 %. The dimensions of the colon and the location and length of the artificial mesenteries were approximately based on reported intraoperative measurements [20].

Testing was carried out in two porcine colons which had been frozen for storage, then thawed to room temperature and cleaned before use. The porcine colons were approximately 120 cm long, with a diameter of 26–35 mm.

Before each test replicate, the CV was lubricated with mineral oil; the efficacy of oil as a lubricant in colonoscopy has been demonstrated in previous clinical trials [21].

### Measurements

Three colonoscopic systems were tested: (1) the HC with automatic control, (2) the HC with manual control and (3) the CC, operated by an experienced colonoscopist (as control). Testing involved advancing the device through the colon in the test bed from the anus to the caecum, the time required to achieve this is described as “insertion time”. Withdrawal of the device and examination of the colon were not included. For each device, we carried out three replicate experiments in each of the two colons tested, for a total of six experiments per device. Reported pressures have been adjusted to represent gauge pressure at the lowest point in the colon, which in our test bed was the anus. This corresponds to the maximum pressure in the colon as pressures at higher points will be reduced due to hydrostatic pressure variation. Forces measured by the load cells were filtered to remove high-frequency noise. Maximum force represents the highest force measured by any of the three load cells.

## Results

The experiments confirmed that it was possible to successfully navigate through the test bed as far as the caecum using the HC. The CC was also successfully inserted.

As the two porcine colons used were not identical, an analysis of covariance (ANCOVA) with planned contrasts was carried out to account for colon-related effects. This colon covariate had a significant effect on the maximum force and the insertion time of the procedures [*F*(1, 16) = 6.92, *p* = 0.02, *r* = 0.55; *F*(1, 16) = 9.99, *p* < 0.01, *r* = 0.62].

The results revealed that the CC applied a significantly higher maximum force to the colon than the HC [*t*(14) = 3.46, *p* < 0.01, *r* = 0.65; CC: 2.22 ± 1.62 N^1^, HC: 0.63 ± 0.41 N], while the HC caused greater pressures at the anus than the CC [*t*(14) = 10.14, *p* < 0.01, *r* = 0.93; CC: 1.53 ± 0.62 kPa, HC: 4.53 ± 0.47 kPa]. No other significant differences were identified. See Table 1 for details.

**Table 1:** Statistics: results summary of the comparison of HC versus CC

	Device		ANCOVA results		
	CC (*N* = 6)	HC (*N* = 12)	*t*(14)	*p*	*r*
Insertion time (min)	4.91 ± 3.28	3.95 ± 3.02	0.82	0.43	0.20
Max. force (N)	2.22 ± 1.62	0.63 ± 0.41	3.46	0.004	0.65
Max. anal pressure (kPa)^a^	1.53 ± 0.63	4.53 ± 0.47	10.14	1 × 10^−7^	0.93
Max. caecal pressure (kPa)	1.52 ± 0.68	2.52 ± 1.25	1.75	0.10	0.40
Mean anal pressure (kPa)	0.65 ± 0.32	1.58 ± 0.46	4.76	0.0003	0.77
Mean caecal pressure (kPa)	0.65 ± 0.36	0.48 ± 0.31	1.07	0.30	0.26

Values under the device columns are mean ± standard deviation

^a^1 kPa = 7.5 mmHg

The comparison of manual and automatic control modes for the HC showed that the automatic controller had a significantly longer insertion time than the manual control mode [*t*(14) = 2.7, *p* < 0.05, *r* = 0.56; automatic: 5.78 ± 2.88 min, manual: 2.11 ± 2.32 min]. However, it also generated significantly lower mean pressures at the anus [*t*(14) = 2.46, *p* < 0.05, *r* = 0.52; automatic: 1.31 ± 0.37 kPa, manual: 1.86 ± 0.42 kPa] and in the sealing balloon [*t*(14) = 2.62, *p* < 0.05, *r* = 0.55; automatic: 1.20 ± 1.61 kPa, manual: 3.87 ± 1.83 kPa]. No other significant differences were identified. See Table 2 for details.

**Table 2:** Statistics: results summary of the comparison of manual versus automatic HC

	Device		ANCOVA results		
	Manual HC (*N* = 6)	Auto HC (*N* = 6)	*t*(14)	*p*	*r*
Insertion time (min)	2.11 ± 2.12	5.79 ± 2.63	2.70	0.02	0.56
Max. force (N)	0.63 ± 0.53	0.63 ± 0.22	0.01	0.99	0.00
Max. anal pressure (kPa)	4.46 ± 0.50	4.60 ± 0.42	0.40	0.69	0.10
Max. caecal pressure (kPa)	2.10 ± 1.03	2.95 ± 1.31	1.29	0.22	0.31
Max. balloon pressure (kPa)	7.44 ± 1.11	7.72 ± 1.02	0.55	0.60	0.14
Mean anal pressure (kPa)	1.86 ± 0.39	1.31 ± 0.34	2.46	0.03	0.52
Mean caecal pressure (kPa)	0.36 ± 0.15	0.60 ± 0.38	1.29	0.22	0.31
Mean balloon pressure (kPa)	3.88 ± 1.67	1.20 ± 1.47	2.62	0.03	0.55

Values under the device columns are mean ± standard deviation

The greatest forces observed at any point during testing were 4.3 and 1.6 N for the CC and HC, respectively. The greatest intraluminal pressures observed during testing were 2.3 and 7.8 kPa for the CC and HC, respectively.

**Fig. 6:**
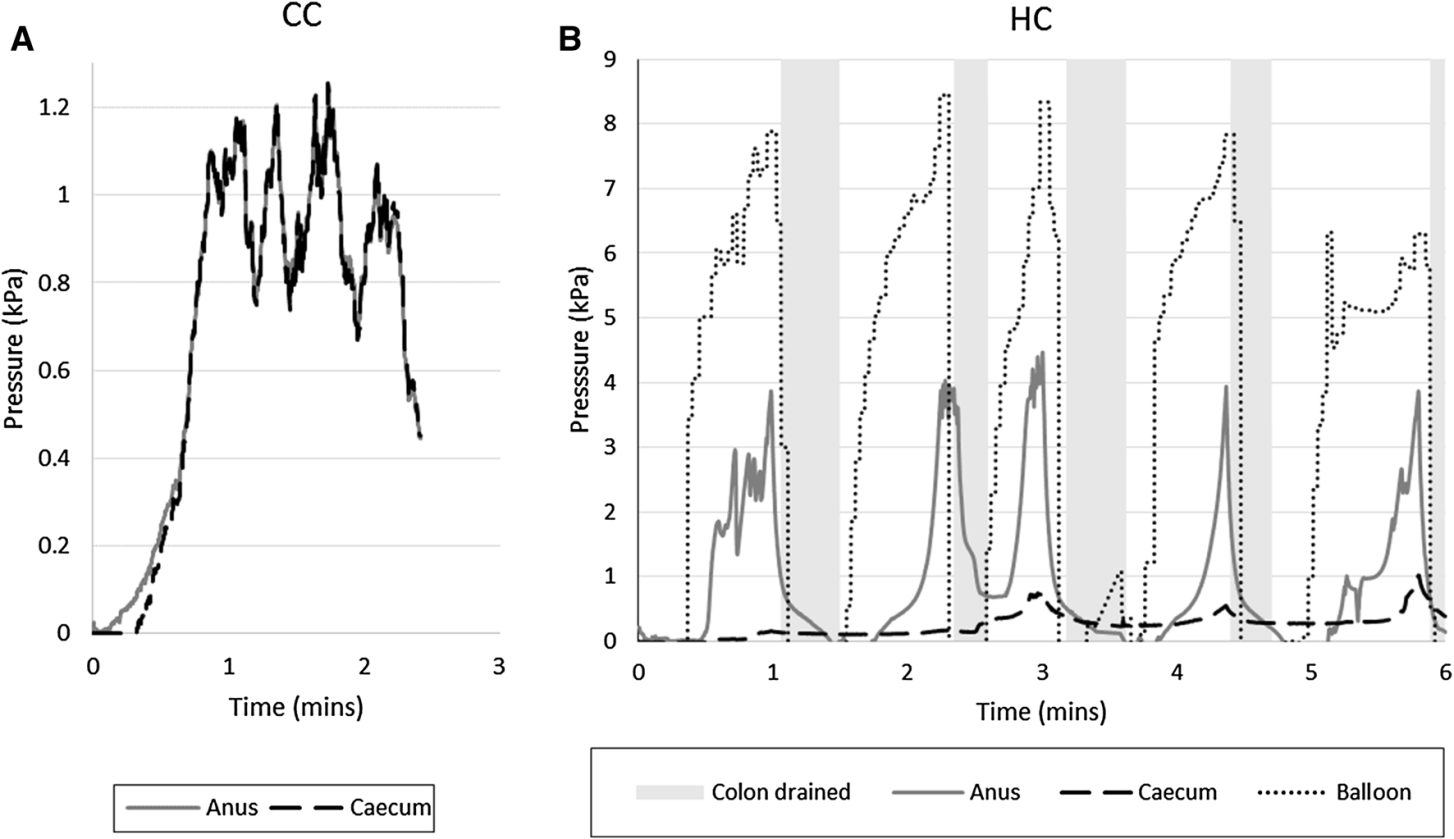
Examples of pressure variation while advancing a CC and automatically controlled HC to the caecum. Pressure is held at a level sufficient to open the colonic lumen during the procedure

**Fig. 7:**
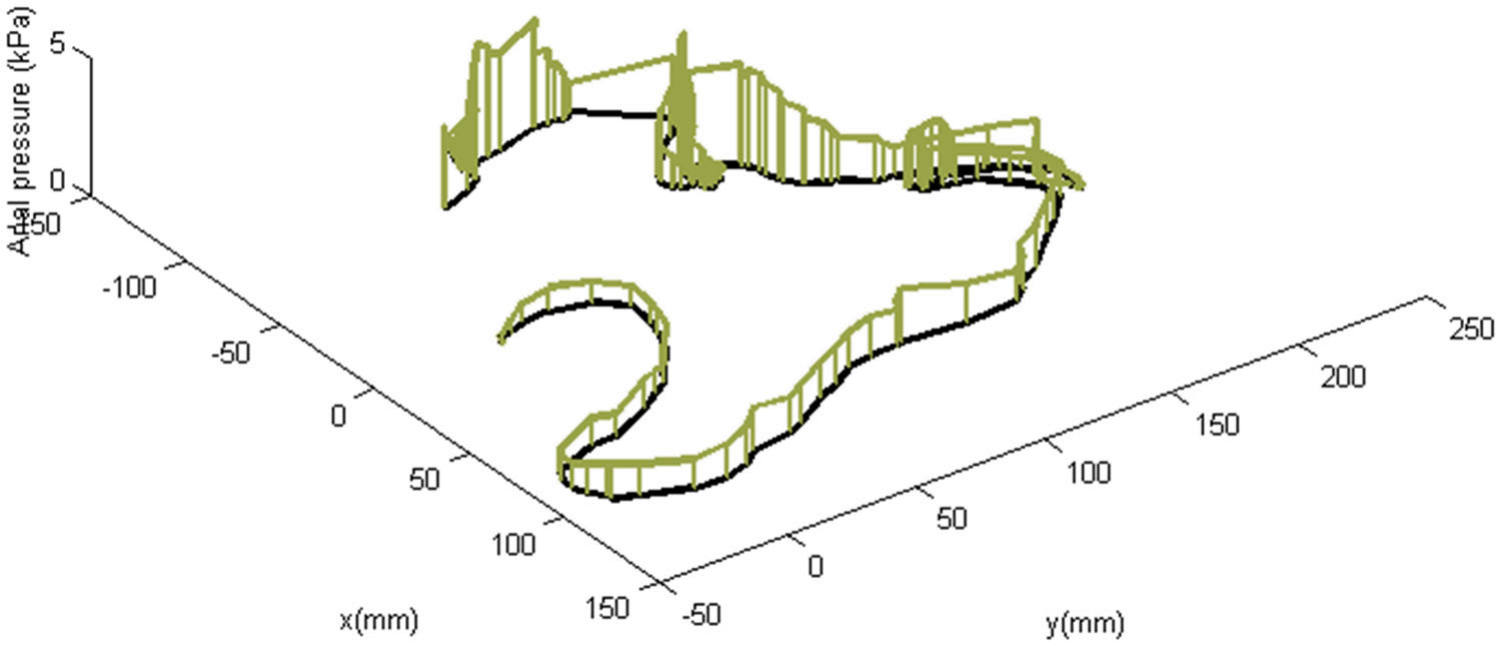
Example of variation of driving pressure and speed with CV position while using the HC with a manual controller. Vertical lines represent pressure and are plotted every second so that their density is inversely proportional to speed

Figure 6 provides details of the intraluminal pressures during one test replicate for the CC and HC, respectively. Note that the pressure in the balloon is partially contained by the balloon itself, so that the pressure exerted on the colon is lower than this value. Pressure behind the CV generally increases until the CV starts to move or pressures become unacceptably high, triggering the draining of the colon. Figure 7 outlines the path of the CV, together with the anal pressure during one test repetition. It demonstrates that pressure generally increases as the CV progresses, presumably due to increasing tether drag, increasing to a maximum when the CV is temporarily stuck in sharp bends or flexures.

## Discussion

### Limitations of the study

Although the shape of the colon was considered to be reasonably realistic, it is not representative of the full range of human colons because of the large natural variation of colonic anatomy in vivo.

The artificial mesentery constraints were placed in anatomically realistic positions, but were unrealistic in that they were discrete rather than a continuous membrane. The forces measured by the load cells were reduced by the friction of the colon on the base and of the cord on the guide. A large fraction of the forces acting on the devices came from the fixed walls of the simulated abdominal cavity, where they could not be measured. For these reasons, we acknowledge that the results may not accurately reflect loads on real mesenteries. Nonetheless, they should allow a reasonable comparison between devices.

Our CC insertion time of 4.9 ± 3.3 min was somewhat shorter, but not significantly different from reported mean clinical times of 6.9 ± 4.2 min [22] (*p* = 0.2). The insufflation pressures used during CC insertion (mean 1.52 kPa) were also low, but within the reported range of clinical values of 1.1–7.6 kPa (mean 3.0 kPa) [23]. The lack of resistance from adjacent anatomical structures may have reduced the pressures required to distend the colon. The similarity of these values to clinical times indicates that the test bed was reasonably realistic.

The colon used was an ex vivo porcine colon. Porcine colon is considered to be morphologically and physiologically similar to human colon [24], but it has a different anatomical layout. The porcine colon had a diameter comparable to sections of human colon, and is a reasonable and far more accessible alterative to in vivo testing. Alternatives such as Thiel-embalmed colon and artificial phantoms were considered to be unsuitable due to their less realistic mechanical and friction characteristics.

The number of test replicates was low (two colons, each with three replicates per device). This was due to the complexity of setting up the test bed and experimental apparatus with fresh colon tissue. Additionally, it was only possible to have a limited number of replicates in each colon due to the fact that the porcine colons tended to deteriorate or ultimately rupture after prolonged testing, limiting their effective lifetimes. We accounted for the low number of colon specimens in our statistical analysis by including the colon as a covariate in an analysis of covariance. While this testing was sufficient for an initial feasibility study, further testing would be required to reliably prove the safety and efficacy of the device.

### Assessment of discomfort

The cause of pain during colonoscopy is excessive strain in the colon and mesocolon; contact with the bowel wall cannot be directly perceived due to the lack of sensory receptors [25] and so is not a direct cause of pain. Due to the ex vivo nature of the experiments, we were unable to assess pain; however, intraluminal pressures measurements and loading of the mesocolon were used as an indirect indicator of discomfort. While it is not possible to say whether a given load or pressure will cause pain, previous reports give an indication of acceptable values. Thus, in a study of insufflation for CT colonography, approximately 50 % of patients experienced pain with insufflation pressures of 5.1 kPa [26]. Pressures of around 3.3 kPa are routinely used in CT colonography, usually without requiring sedation [27]. For mesentery loading, a colonoscopic device was reported to cause peak loads on an artificial mesentery of around 1 N compared to a CC which caused peak loads of around 4.5 N. In a clinical trial, this device was given an average pain score of 1/10, while a CC scored 6.9/10 (higher values are more painful) [15].

Patients’ post-procedure perception of pain during colonoscopy has been reported to more closely correlate with the instantaneous maximum pain experienced than the time-averaged pain [28]; therefore, peak values of force and pressure are of particular interest. Our experiments showed average peak pressures of 4.5 kPa for the HC, compared to 1.5 kPa for the CC, and peak mesentery loads of 0.6 N for the HC, compared with 2.2 N for the CC. Therefore, we may expect that the HC will reduce pain due to mesentery loading, but increase pain due to intraluminal pressure when compared to the CC.

### System safety

The average burst pressure of a cadaveric human colon is reported to be approximately 15 kPa, and the observable trauma due to pressure occurs at ≥6.9 kPa [23]. Burst occurs first at the caecum, which is the area that is least pressurised by the HC. However, as living patients will have tone in the colonic muscular coats, it is probable that higher pressures are needed to cause trauma and perforation in vivo. Sustained intraluminal pressures of up to 6.5 and 7.6 kPa have been reported during CT colonography and colonoscopy, respectively [23, 26]. Therefore, the HC with mean peak pressures of 4.5 kPa utilises pressures within the range used in current clinical practice. These pressures were below those expected to cause damage or colonic perforation, but the margin of safety is small. During the experiments, the forces applied by the HC to the mesentery were lower than those generated by the conventional colonoscope (0.63 vs 2.22 N) and so are considered to be safe.

### Comparative performance between HC and expert colonoscopy

The insertion times were comparable between the CC and HC, and differences were not statistically significant. Mesentery loading was significantly less with the HC, this may be explained by the fact that the HC is designed to be flexible and has approximately one hundred times less flexural rigidity than a CC (roughly 2 vs 200 N cm^2^ [29]).

The HC is currently a simple device, with reduced capabilities compared to a CC; it does not have a steerable tip, and in its present form, it does not possess capability for biopsy. However, its simplicity has advantages in that it is easy to produce, control and automate.

Although the current prototype contains a rigid cylinder to simulate carrying an on-board camera, no camera has been installed. As a result, we are not able to assess the quality of imaging acquired. As the tip is not actively steerable, the ability to closely examine a given anatomical feature is limited. However, with a wide-angle lens, it should be possible to inspect the colon. It was observed that the tip of the CV passively tends to point along the lumen of the colon; however, it may touch the colonic wall in tight flexures, potentially causing “red-out” as happens in routine manual colonoscopy.

### Choice of driving fluid

The experiments have confirmed that water is a viable driving fluid, in contrast to the Aer-O-Scope which uses CO_2_ and is of proven clinical viability. Both fluids have merits. CO_2_ is around 100 times less viscous than water, which can allow for a smaller, more flexible device to be constructed. Additionally, it is easier to remove fluid from in front of the CV; if a tube of reasonable diameter is provided gas should flow out naturally, whereas water requires a suction pump to drain the colon. A second advantage of using a gas is that hydrostatic pressure differentials become negligible; this will reduce the maximum pressure acting on the colon for a given mean diving pressure.

In contrast, use of warm water helps to relax the patient’s bowel, improving patient comfort [16, 30]. As a more viscous fluid, water has less tendency to leak past the seal. This advantage is important as high leak rates can rapidly reduce pressure differentials across the CV, making motion less reliable. Our testing indicated that when using oil for lubrication, the difference in friction between using air and water as a driving fluid is small.

## Conclusion

We have demonstrated the ability of a HC to navigate an in vitro test bed, which was modelled on a human colon. A comparison to a standard colonoscope showed that the HC caused reduced loading on the artificial mesenteries, but caused increased intraluminal pressures in the lower colon. The data indicate that based on strain applied to the colon and mesocolon, the device could reduce patient discomfort.

While it has previously been demonstrated [12] that a pressure-driven colonoscopic device can navigate the colon, we have demonstrated that the same can be achieved using water, using driving pressures comparable to those used in current clinical practice.

Finally, we have also shown that the HC can be used under automatic or manual control. A finite state machine-based automatic controller was developed which was able to successfully navigate the test bed based on feedback from pressure and movement sensors. The automatic controller was able to successfully navigate the colon with lower mean pressures in the lower colon and sealing balloon than used by a human controller. However, the automatic controller was has a significantly longer insertion time than manual control. As the HC can be driven by an automatic controller, it has the potential to reduce the amount of skill required by the operator to advance the device to the caecum.

## Footnotes

1 All values are mean ± SD.
